# Single-Step Fast Tissue Clearing of Thick Mouse Brain Tissue for Multi-Dimensional High-Resolution Imaging

**DOI:** 10.3390/ijms23126826

**Published:** 2022-06-19

**Authors:** Youngjae Ryu, Yoonju Kim, Hye Ryeong Lim, Hyung-Joon Kim, Byong Seo Park, Jae Geun Kim, Sang-Joon Park, Chang Man Ha

**Affiliations:** 1 Research Strategy Office and Brain Research Core Facilities of Korea Brain Research Institute, Daegu 41068, Korea; ryj123@kbri.re.kr (Y.R.); pray4u96@kbri.re.kr (Y.K.); hrsz@kbri.re.kr (H.R.L.); 2 Department of Histology, College of Veterinary Medicine, Kyungpook University, Daegu 41566, Korea; psj26@knu.ac.kr; 3 Dementia Research Group, Korea Brain Research Institute, Daegu 41068, Korea; kijang1@kbri.re.kr; 4 Division of Life Sciences, College of Life Sciences and Bioengineering, Incheon National University, Incheon 22012, Korea; bbs0808@naver.com (B.S.P.); jgkim@inu.ac.kr (J.G.K.)

**Keywords:** deep brain tissue, tissue clearing, refractive index matching solution, molecular imaging, low viscosity, simple immersion, light sheet fluorescence microscopy

## Abstract

Recent advances in optical clearing techniques have dramatically improved deep tissue imaging by reducing the obscuring effects of light scattering and absorption. However, these optical clearing methods require specialized equipment or a lengthy undertaking with complex protocols that can lead to sample volume changes and distortion. In addition, the imaging of cleared tissues has limitations, such as fluorescence bleaching, harmful and foul-smelling solutions, and the difficulty of handling samples in high-viscosity refractive index (RI) matching solutions. To address the various limitations of thick tissue imaging, we developed an Aqueous high refractive Index matching and tissue Clearing solution for Imaging (termed AICI) with a one-step tissue clearing protocol that was easily made at a reasonable price in our own laboratory without any equipment. AICI can rapidly clear a 1 mm thick brain slice within 90 min with simultaneous RI matching, low viscosity, and a high refractive index (RI = 1.466), allowing the imaging of the sample without additional processing. We compared AICI with commercially available RI matching solutions, including optical clear agents (OCAs), for tissue clearing. The viscosity of AICI is closer to that of water compared with other RI matching solutions, and there was a less than 2.3% expansion in the tissue linear morphology during 24 h exposure to AICI. Moreover, AICI remained fluid over 30 days of air exposure, and the EGFP fluorescence signal was only reduced to ~65% after 10 days. AICI showed a limited clearing of brain tissue >3 mm thick. However, fine neuronal structures, such as dendritic spines and axonal boutons, could still be imaged in thick brain slices treated with AICI. Therefore, AICI is useful not only for the three-dimensional (3D) high-resolution identification of neuronal structures, but also for the examination of multiple structural imaging by neuronal distribution, projection, and gene expression in deep brain tissue. AICI is applicable beyond the imaging of fluorescent antibodies and dyes, and can clear a variety of tissue types, making it broadly useful to researchers for optical imaging applications.

## 1. Introduction

Three-dimensional volumetric imaging of biological tissue is critical for uncovering intrinsic morphology and biological information, as well as pathological changes during disease. Pathological changes may include alterations in tissue structure, cellular components, functional responses to the environment, and tissue networks. However, volumetric imaging is limited when it comes to deep tissues and high-resolution image acquisition as most samples are opaque. The resulting light scattering and absorption, as well as autofluorescence, limit the imaging of complex organ samples [1]. Two-photon microscopy is useful for deep tissue imaging, but spherical aberration effects still limit accurate image capture [2]. Moreover, the opacity of fixed brain tissue limits deep imaging to approximately 300 μm, even if two-photon microscopy is being used [3]. Recent optical imaging techniques can capture data to a depth of several millimeters in the mouse brain by using scattered light [4,5] and compensating for optical aberrations [6,7]. However, these optical techniques still have problems obtaining high-resolution, deep images of biological samples due to tissue scattering and different refractive index (RI) distribution in the tissue.

To overcome these limitations, optical clearing methods have been developed that involve lipid removal and replacing the sample solution with one that matches the refractive index (RI) of the tissue. These optical clearing techniques fall into four families based on the underlying physicochemical method used: immersion in an organic solvent [8,9,10,11,12,13,14]; protein hyperhydration [15,16,17,18,19]; tissue transformation with hydrogel embedding [20,21,22]; and simple immersion in high RI solutions [3,23,24,25,26,27,28]. The solvent-based methods occur the sample shrinkage and the quenching of fluorescent protein emission through a dehydration step, and with a need to change the skin toxicity solution for the additional tissue clearing (~2 h), storage, and imaging solution (e.g., dibenzyl ether). Other optical tissue clearing methods also ultimately require a high RI matching solution to reduce the spherical aberration of thick tissue that compensates for the light-distorting effects of the coverslip and the immersion oil used for the objective lens (RI 1.51) [1,29].

Cleared samples using passive immersion clearing methods can be directly applied to microscopic imaging since they simultaneously match RI and clear tissue using a range of optical clear agents (OCAs). OCAs containing glycerol [30] and saccharides, such as fructose [23,25] and sucrose [3], require high concentration solutions to match high RIs. This leads to the high viscosity of the resulting OCA solution, making it difficult to work with. High-concentration OCAs with low viscosity, such as 2,2′-thiodiethanol (TDE) [26,29] and formamide [27], also reduce the fluorescence signal and cause tissue hardening. Hence, alternative immersion clearing solutions such as FocusClear [31] and refractive index matched solution (RIMS) [21] were developed based on contrast agents, including diatrizoic acid, histodenz, and other complex molecules. Alternative immersion solutions are now commercially available and include EasyIndex (LifeCanvas Technologies, Cambridge, MA, USA), XclarityMS (Logos Biosystems, Gyeonggi-do, Korea), and MS (BINAREE Inc., Daegu, Korea). Protocols for using these solutions generally use the RI matching solution as a last step as there is insufficient tissue penetration without a lipid clearing step. Therefore, the relative efficiency of these simple immersion solutions in terms of RI matching speed, clearing depth for intact tissue, and morphology distortion remains unclear. Furthermore, these commercial RI matching solutions have critical issues, including high viscosity, the introduction of air bubbles, precipitation, crystallization, and drying out. These issues limit the utility of these solutions in sample preparation for confocal microscopy and light sheet fluorescence microscopy (LSFM).

Here, we developed a new Aqueous high refractive Index matching and tissue Clearing solution for Imaging (AICI) for easy application to microscopic imaging. AICI can rapidly clear tissue: it can clear a 1 mm-thick brain slice within 90 min at 35 °C. The viscosity of AICI is close to that of water and it remains fluid, even after exposure to air for long intervals. This means that AICI can be used during the long periods needed for imaging thick tissue samples. Changes in the linear morphology of brain tissue are less than 2.3% over 24 h, meaning that the dimensions of tissue samples are broadly preserved. AICI allows the imaging of fine structures, such as dendritic spines and axon boutons, in a deep brain slice. Thus, a short period of clearing with AICI enables large-field three-dimensional (3D) analysis of the synaptic network. These properties of AICI, such as low-viscosity, high-refractive-index matching, and low morphological distortion, make it useful for a broad range of imaging applications.

## 2. Results

### 2.1. Limitations in Three-Dimensional Imaging for Cleared Tissue

**Figure 1:**
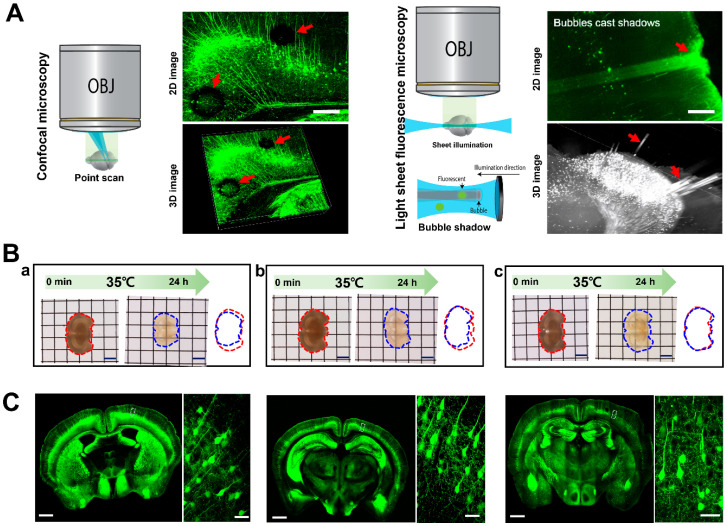
Technical limitations in multidimensional imaging and development of AICI solution. (**A**) Air bubbles on the sample surface or inside tissue caused aberrations in imaging using light microscopy. Multidimensional images by fast confocal microscopy (upper panel) and Lightsheet Z.1 (lower panel) show that bubbles impede the 2D and 3D rendering of images in cleared brain tissue by CLARITY. Red arrows represent bubble obstacles. Scale bars, 200 µm. (**B**) Comparison of tissue transparency and distortion by reagent change. The 1 mm-thick brain slice was treated with different components of the AICI solution. (a) Brain slice treatment with 10% (wt/vol) *N*-methyl-D-glucamine, 17% (wt/vol) D-Sorbitol, 22% (vol/vol) TDE, 0.5% (wt/vol) saponin, 1% (vol/vol) Triton X-100, 0.5% (wt/vol) α-thioglycerol, and 0.025% (wt/vol) NaBH4 for 90 min. Refractive index was 1.463. (b) Brain slice treatment with 20% (wt/vol) *N*-methyl-D-glucamine, 30% (vol/vol) TDE, 45% (wt/vol) Iodixanol, 0.2% (wt/vol) Triton X-100, 0.5% (wt/vol) α-thioglycerol, 0.1% (vol/vol) triethanolamine, and 0.25% NP-40 (vol/vol) for 90 min. Refractive index was 1.472. (c) The c solution substituted 1% (wt/vol) saponin for 0.25% NP-40 (vol/vol) and treated the brain slice for 90 min. Refractive index was 1.468. Right panel of each image indicates the sample area, the red-dotted line represents the border of the initial brain slice at 0 min, and the blue-dotted line represents the border of brain slices treated with a different OCA component of the AICI matching solution after 90 min. Scale bars, 3 mm. (**C**) Representative photographs showing the coronal section of Thy1-GFP-M mouse brain in each OCA component solution of panel B, respectively. Inset of each image shows high-magnification images. Scale bars, 50 µm.

Various optical clearing methods promise a greater understanding of the nervous system through large volumetric imaging of the brain. However, blurry z-axial depth volume images occur due to insufficient tissue clearing and different RI immersion solutions. Image blurring results in incorrectly reconstructed images, preventing the accurate 3D rendering of the cleared tissue. Confocal microscopy has advantages in that it decreases the blurriness of images by increasing signal-to-noise ratios and decreases the cross-excitation of channels in multiple-color-labeled brain tissue. However, confocal microscopy still has several limitations. For example, confocal imaging can be limited by air bubbles inside or outside the sample during the tissue clearing and high viscosity RI matching process, a slow scan speed, and photobleaching of the sample by point scanning (Figure 1A, left panel). LSFM can overcome limitations in the time required to produce large brain section images, thus reducing photobleaching compared with confocal microscopy. UltraMicroscope II and UltraMicroscope Blaze (Miltenyi Biotec, Bergisch Gladbach, Germany) and Lightsheet Z.1 and Lightsheet7 (Carl Zeiss, Jena, Germany) are commercially available LSFMs. However, LSFM imaging is also distorted by air bubbles, causing light shadows in 2D imaging and a cylindrical shape in 3D volumetric images (Figure 1A, right panel). The major impediments to 3D volume imaging are sample distortion due to expansion during the clearing process and air bubble formation in the high viscosity RI matching solution. To address these issues, we developed an AICI solution that avoids the introduction of air bubbles for multidimensional imaging, minimizes sample distortion, and facilitates tissue clearing. To optimize the AICI solution composition, we compared the different chemical compositions of RI matching solutions with 1 mm thick, 4% paraformaldehyde-fixed mouse brain slices for 24 h at 35 °C, and the tissue transparency was similar within 24 h of incubation (Figure 1B). However, the brain slice distortion was dramatically reduced by the OCA composition in Figure 1B(c). The dendrite fluorescent intensity was decreased by the OCA composition in Figure 1B(a), whereas the fluorescent intensity of the dendrite and cell bodies was well maintained by the OCA composition in Figure 1B(b,c),C. Therefore, we used the OCA composition in Figure 1B(c) as the OCA composition of AICI, and further identified the appropriate temperature for tissue clearing of the AICI solution. AICI rapidly penetrated and cleared the sample with treatment times ranging from 30 min to a few hours for the brain slices at both 25 °C and 35 °C, and the clearing ability at 35 °C was improved compared with the brain slices immersed at 25 °C (Supplemental Figure S1).

### 2.2. AICI Enables Rapid Tissue Clearing and Low Sample Distortion

**Figure 2:**
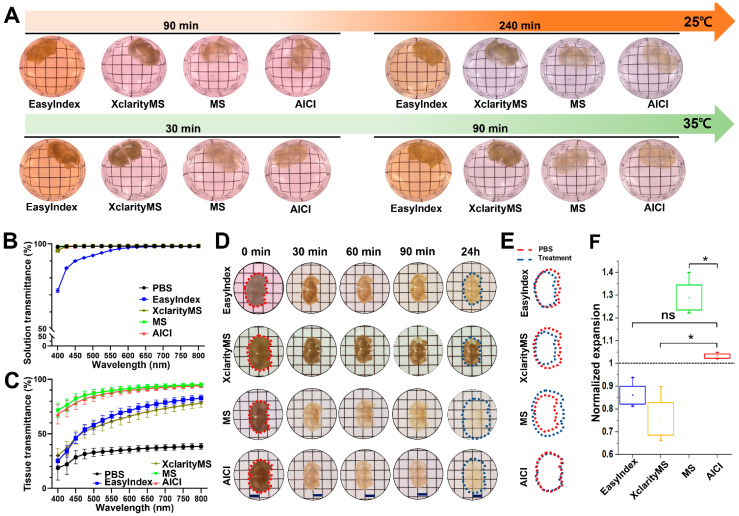
Comparison of tissue transparency and distortion in AICI with other OCA-based commercial RI matching solutions. (**A**) Representative images of tissue transparency at different temperatures (25 °C, 35 °C) in AICI with other OCA-based commercial RI matching solutions. The 1 mm thick coronal brain slices from Thy1-EGFP mice were treated with each RI matching solution and imaged at the indicated time points. (**B**) Transmission curves of each indicated RI matching solution (mean ± SEM, *n* = 3 measurements). (**C**) Transmission curves of the 1 mm brain slices treated with each indicated RI matching solution. Transmittance was measured along the cortex part of the 1 mm brain slices after 1 h incubation at 35 °C. Data are presented as the mean ± SEM (*n* = 3 brain slices). (**D**) Representative images of tissue distortion at the indicated time points (0, 30, 60 min and 24 h) at 35 °C in each RI matching solution. Scale bar, 3 mm. (**E**) The red-dotted line represents the border of the initial brain slice at 0 min, and the blue-dotted line represents the border of brain slices treated with each RI matching solution after 24 h. (**F**) Normalized deformation of brain slices after treatment with each RI matching solution. Sample distortion was represented relative to the initial brain slice outlines before (blue dots) and after immersion for 24 h (red dots) in each RI matching solution. The box-whisker plot indicates the mean ± SEM (*n* = 4). Statistical significance (* *p* < 0.05) was assessed by one-way ANOVA with Tukey tests.

Various high-viscosity sugar solutions have been used as RI matching solutions [3,23]. In addition, *N*-methyl-D-glucamine and iodixanol solution have been used as high RI matching and tissue clearing solutions when imaging brain samples [32]. TDE (2,2′-thiodiethanol) has also been used with a water-soluble mounting medium for high-resolution imaging deep inside fixed specimens [26,29]. We used 47% TDE for the AICI solution, and identified that only 47% TDE was insufficient for tissue clearing speed, depth penetration, and 3D depth images compared with the complete AICI composition (Supplemental Figure S2). AICI also contains OCAs and contrast agents, such as iodixanol, and is competitive in both performance and price compared with other commercial RI matching solutions. In brief, AICI contains a low concentration of TDE that prevents a reduction in fluorophore brightness [29], and *N*-methyl-D-glucamine as a high RI solution instead of high concentrations of sugar or fructose. The nonionic detergent Triton X-100 is a hydrophilic, mild surfactant that does not denature proteins [33]. Therefore, a low concentration (0.2%) of Triton X-100 was included to increase cell permeability. We used 2,2′,2″-nitrilotriethanol/triethanolamine as another surfactant in AICI as it can remove fatty acid and oil in tissues, and α-thioglycerol was used to avoid the browning and autofluorescence of the AICI solution during incubation at 35 °C [34]. AICI was developed as an aqueous-based high RI matching solution for use in multidimensional imaging. AICI solution has a final RI (1.466 ± 0.012) at both 25 °C and 35 °C. Immersion OCAs usually cause large sample volume changes and loss of color [35]. To compare with AICI properties, we performed a quantitative inspection of brain slices with different OCA-based commercial RI matching solutions. EasyIndex (RI 1.468 ± 0.012) and XclarityMS (RI 1.462 ± 0.012) did not clear the brain slices, even after a 36 h immersion, whereas AICI and MS (RI 1.461 ± 0.015) rapidly cleared the 1 mm thick brain slices (Figure 2A) during the initial 30 min at a temperature of 35 °C. To evaluate the tissue-clearing ability of the AICI solution, we compared the light transmittance in the cortex region of adult mouse (postnatal day 60) brain slices with different OCA-based RI matching solutions. The AICI solution did not interfere with transmittance at different wavelengths, and the AICI- and MS-incubated mouse brain slices were more transparent than was the case with other RI matching solutions under the light-wavelength range of 400–800 nm (Figure 2B,C).

We further measured volume changes in the brain slices in different RI matching solutions. All the RI matching solutions slightly decreased the sample volume after 90 min (Figure 2D). We then quantified the sample volume changes at 24 h. AICI produced only a 2.3% tissue linear expansion in the sample after immersion compared with the sample dimensions in the phosphate-buffered saline (PBS) solution at the start of the experiment (0 min). However, there was a 14.4% shrinkage with EasyIndex, a 24.7% shrinkage with XclarityMS, and 28.6% expansion with the MS solution compared with the PBS solution immersion (Figure 2E,F). These results show that AICI can preserve tissue morphology with rapid RI matching and tissue clearing.

### 2.3. AICI Is an Aqueous, High-Refractive-Index Solution with Low Viscosity

**Figure 3:**
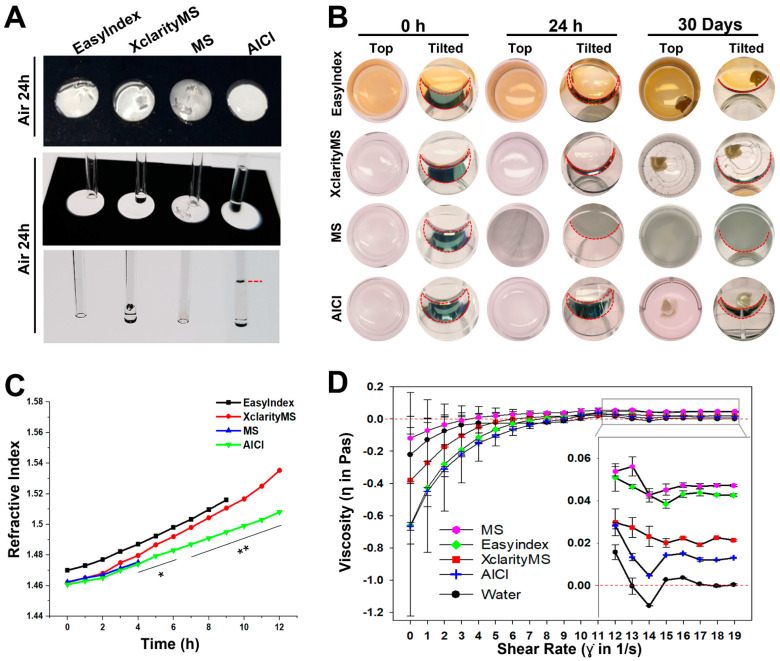
Fluid flow characteristics of each RI matching solution. (**A**) Representative images of the comparison of capillary action after exposure to air for 36 h for each RI matching solution. AICI was observed at the highest (red-dotted) line. (**B**) The hardening process of each RI matching solution in a 24-well plate at 25 °C for 24 h to 30 days. Top shows each RI matching solution contained in the well plate when the plate was laid flat, and when the well plate was tilted. The flow of RI solution is outlined with dashed red lines. Only the AICI solution was retained in a liquid state, whereas other matching solutions hardened and crystallized after immersion for 30 days. (**C**) The refractive index changes are time dependent. An amount of 500 µL of each RI matching solution was placed in a 24-well plate with air exposure in a laboratory environment and measured for refractive index using an Abbe refractometer (NAR-1T solid, ATAGO, Tokyo, Japan). The refractive index displaying the dotted graph for each RI matching solution and statistical significance (** *p* < 0.01, * *p* < 0.05) was assessed by one-way ANOVA with Duncan’s test (*n* = 3). (**D**) The viscosity of each reagent was measured by a rotational rheometer system. Insets are enlarged to distinguish the viscosity data of each reagent following step 12. The viscosity of the AICI reagent is closest to that of water (20 steps). Data plot indicates the mean ± SEM (*n* = 3).

**Figure 4:**
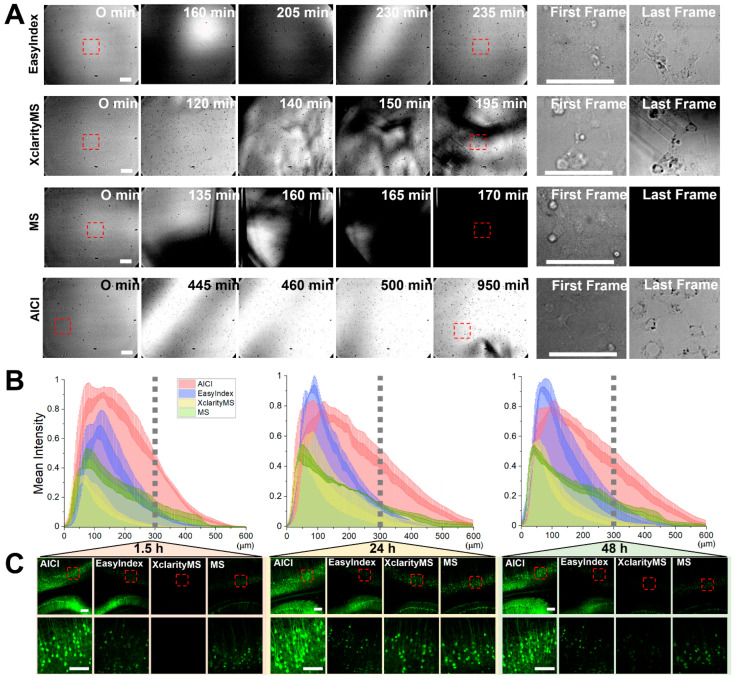
Comparison of long-term imaging and fluorescence preservation in different RI matching solutions. (**A**) 4% PFA-fixed HEK293T cells in a 24-well cover glass chamber were imaged for morphological changes over time with 500 μL of each RI matching solution using a Nikon perfect focus system (PFS). The cells on the glass bottom in the red inset box represent enlarged images (first and last frame, right). Scale bars, 50 μm. (**B**) Z-plot profiles show changes in fluorescence intensity in 600 μm of Thy1-GFP-M mouse brain slice by tissue penetration of each RI matching solution. The fluorescence intensity was normalized to the peak value by each RI solution using a confocal microscope with a 10× objective. AICI rapidly penetrated the brain slice tissues and the fluorescence intensity was highly preserved by AICI, rather than the other reagent-based RI matching solutions. (**C**) Representative images of fluorescence intensity at a median depth (z = 300 µm, grey-dotted lines in (**B**)) of hippocampal CA1 and cortical regions treated with each RI matching solution at indicated times. Scale bar, 200 μm. Inset scale bar, 100 μm.

We next examined the viscosity of AICI. A well-balanced viscosity would avoid both the formation of air bubbles and the evaporation of the RI matching solution during lengthy multidimensional image acquisition. The capillary tube allows an easy comparison of viscosity by measuring the vertical fluid flow by capillary action. The capillary action after exposure to air for 24 h in each RI matching solution showed that AICI retained its solution status, whereas XclarityMS almost completely evaporated, and EasyIndex and MS dried and solidified after 36 h of air exposure (Figure 3A). An amount of 1 mm of each solution was kept for 30 days in a covered 24-well plate, and only AICI retained its liquid state (Figure 3B). To identify the RI alteration of each matching solution, 500 μL of each RI matching solution was exposed to air in the laboratory environment. All the RIs increased upon evaporation of the matching solution, while MS and EasyIndex failed to measure an RI after 8 h owing to hardness or crystallization. The RI of AICI showed a moderate increase up to 1.508 (±0.001), whereas XclarityMS showed an increased RI up to 1.535 (±0.002). Both the AICI and XclarityMS solutions showed significant differences from 4 h after exposure (Figure 3C). Furthermore, AICI was closest to the viscosity of water, as tested using a rheometer system to compare the viscosity of the RI matching solutions (Figure 3D). Consequently, AICI is able to store a brain sample for at least 30 days while maintaining a low viscosity and a high RI ratio status. This result suggests that AICI can be used for the lengthy imaging of samples with large volumes and long-term sample storage. To evaluate the advantages of AICI compared with other RI matching solutions, we performed overnight imaging of HEK239T cells fixed in 4% paraformaldehyde with each RI matching solution at 25 °C using the Nikon perfect focus system (PFS). The EasyIndex solution started hardening at 160 min, and was completely hardened after 4 h of air exposure. Interestingly, EasyIndex can take cell images after completely solidifying using the PFS, but the cell images are altered compared with the first frame in liquid states. The XclarityMS and MS started to solidify and crystallize after air exposure for 2 h, resulting in changes in cell morphology: the images were altered or out of focus with the PFS. While AICI was slightly dried after air exposure for 445 min, imaging for 15 h was still possible with the PFS, and the cell morphology was well maintained (Figure 4A). We then compared the optical clarity for brain depth and fluorescence preservation in each RI matching solution. We used postnatal 40-day adult Thy-1-GFP line M (Thy1-GFP-M) mouse brains [36] and imaged them with a 10× air Nikon Plan Apo objective lens (N.A = 0.45, working distance 4.0 mm) using confocal microscopy. The resulting images show that GFP fluorescence intensity in AICI according to sample depth reached a peak intensity within the first 90 min, due to the rapid penetration and clearing of the brain slices compared with the other RI matching solutions. The fluorescence intensity of EasyIndex reached a peak after 24 h, but the depth penetration was thin such as XclarityMS. The MS matching solution quickly penetrated the brain slices (as in Figure 2C), but the fluorescence intensity quickly diminished (Figure 4B). The single frame images at 300 μm depth adequately show that AICI deeply penetrated the brain slices and that the fluorescence intensity was well preserved (Figure 4C). We further identified whether the fluorescent signal was preserved over time in the different matching solutions. Approximately 65% of the GFP fluorescence intensity relative to peak GFP fluorescence signals was preserved in AICI after 10 days, whereas the fluorescence intensity in XclarityMS and MS was relatively reduced in intensity at the initial time and at 10 days. In the case of EasyIndex, GFP fluorescence intensity at 10 days was preserved, as in AICI, but was reduced by approximately 48% compared with peak intensity, even though we changed the solution to avoid solidification (Supplemental Figure S3).

### 2.4. AICI Minimizes Reduction of Fluorescence Intensity and Distortion of Neuronal Structure

**Figure 5:**
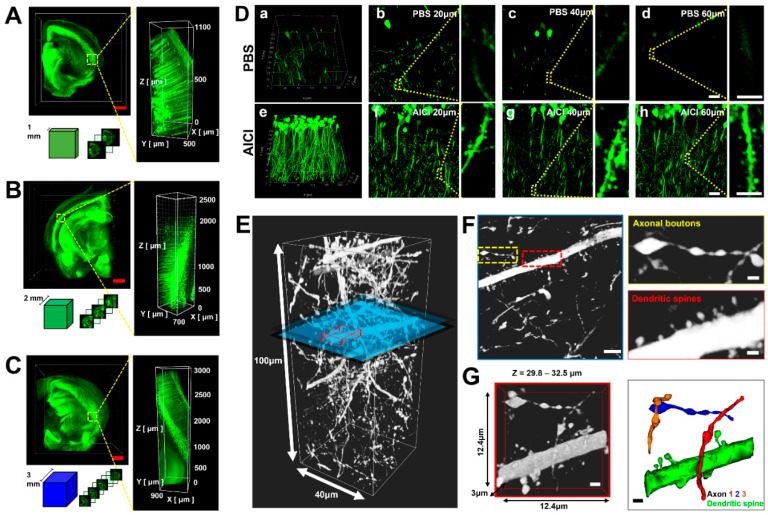
Depth capacity and morphology maintenance of AICI with three-dimensional neuronal imaging. (**A**–**C**) The penetration and clearing capacity of AICI by sample thickness was performed for the indicated immersion times. The three-dimensional limitations of optical imaging were identified by reconstructing images from a 1–3 mm depth of AICI-treated Thy1-GFP-M mouse brain slices using Lightsheet Z.1 with a 5× objective lens. Sectional images at different 3D depths are shown with a 500 μm z-step. Scale bars, 1 mm. (**D**) Comparison images of neuronal structures at representative depths after PBS and AICI clearing using Nikon A1Rsi with 100× Plan Apo Lambda Oil lens (N.A 1.40, WD 0.13 mm). Three-dimensional reconstruction of images of neuronal dendrites in PBS (a) and after AICI clearing for 90 min (e). Section images of neuronal dendrites at the indicated depths in PBS (b–d) and after AICI for 90 min (f–h). Scale bars, 5 μm. (**E**) Thy1-GFP-M mouse brain slice cleared by AICI reconstructed in 3D in the *z*-axis (100 μm and 40 μm x-y plane). (**F**) Image in the blue section in (**E**) shows axonal boutons and dendritic spines after AICI clearing. Right panel shows an enlarged image of the yellow- and red-dotted boxes in the left panel. Scale bar, 5 μm. Inset scale bar, 1 μm. (**G**) 3D reconstruction of detailed neuronal synapse morphology by AICI clearing represented by the red box in panel (**E**). Scale bar, 1 μm.

AICI allowed rapid optical clearing and improved the fluorescence intensity of deep sample imaging compared with commercial RI matching solutions. We next examined the depth of imaging in AICI with Thy1-GFP-M mice brain slices using LSFM (Zeiss Z.1 with 5× objective lens). A 1–3 mm-thick brain slice was easily cleared within 1.5 h, 1 day, and 3 days by AICI, and 3D *z*-axis rendering allowed imaging of the fine neuronal structures through all of the sectioned images (Figure 5A–C). However, we found that AICI did not completely clear 4 mm-thick brain slices, even after incubation for over 3 days at 35 °C. To examine the image quality in AICI, Thy1-GFP-M mice brain slices were imaged at a 60 μm depth with a confocal microscope (Nikon A1Rsi with 60× Plan Apo Lambda Oil lens, N.A 1.40, WD 0.13 mm). The GFP fluorescence intensity of the neuronal dendrites started to disappear at a depth of 40 μm in PBS. An increased background was seen, and the fluorescence intensity was severely reduced at 60 μm in PBS; whereas the details of the neuronal cell bodies and dendrites were well preserved in the whole volume at a depth of 60 μm after AICI clearing (Figure 5D). We further examined whether AICI can preserve fluorescence intensity in fine neuronal structures such as dendritic spines and axons using a high-resolution confocal microscope (Nikon A1Rsi with 100× Plan Apo Lambda Oil lens, N.A 1.40, WD 0.13 mm). The fluorescence intensity in the dendritic spines and axonal boutons was well preserved in a 100 μm deep reconstructed image after AICI clearing (Figure 5E,F), and the synapse networks could be identified through 3D rendering at this depth (Figure 5G).

### 2.5. AICI Is Suitable for Three-Dimensional and Multifunctional Imaging Using Multicolor Fluorescence

**Figure 6:**
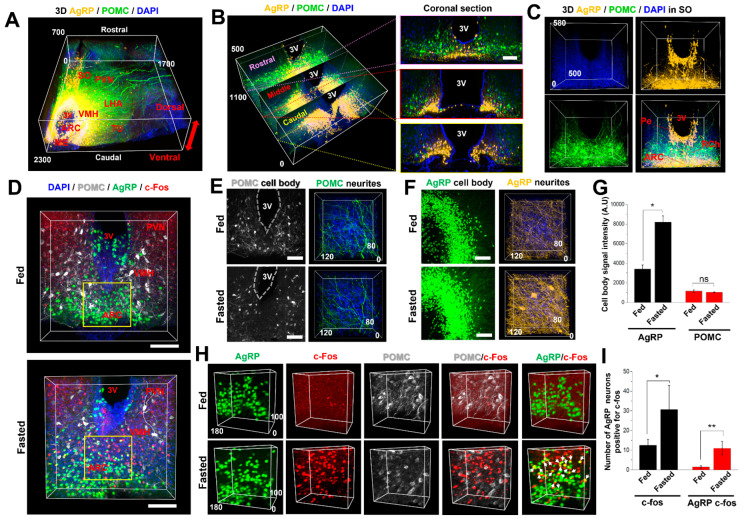
Application of AICI to three-dimensional and functional imaging. (**A**) 3D-reconstruction of AgRP Cre-tdTomato neurons (yellow), POMC-EGFP neurons (green), and DAPI (blue) images for the nucleus in the mouse brain hypothalamus region. SO, supraoptic nucleus; PVN, paraventricular nucleus; LHA, lateral hypothalamic area; VMH, ventromedial hypothalamic nucleus; ARC, arcuate hypothalamic nucleus; ME, median eminence; TU, tuberal nucleus; and 3V, third ventricle. (**B**) Representative 3D volume image from the rostral to caudal ARC region by AICI immersion. Each two-dimensional selected single image was sorted from the 3D-reconstructed image (left panel). Scale bars, 100 μm. (**C**) The 3D reconstructed image of the SO region clearly shows the AgRP cells axially located on the ARC as well as the periventricular hypothalamic nucleus (Pe) of the rostral hypothalamus, whereas the cell bodies of the POMC neurons are predominantly located in the ARC and the retrochiasmatic area (RCh). (**D**) The 3D expression pattern of AgRP (green), POMC (gray), and c-Fos (red) in the hypothalamic ARC region under fed and fasted conditions. Scale bars, 100 μm. (**E**,**F**) The maximum intensity projection images of AgRP, POMC neuronal population, and neurite density in the ARC and VMH regions relatively. Scale bars, 100 μm. (**G**) Bar graphs displaying quantitative analysis of the integrated density of the AgRP and POMC neuronal cell bodies in each experimental group (*n* = 4). (**H**) The 3D-rendered images of AgRP and POMC in the fed and fasted mice easily show the neuronal population, colocalization, and density of neurites. The c-Fos (red) was stained with a primary antibody followed by Alexa Fluor 647 secondary antibody using electrophoretic antibody staining method, as discussed in the materials and methods. The arrow indicates colocalized cell bodies in inset. (**I**) Bar graph displaying the number of AgRP neurons positive for c-Fos in the ARC in each experimental condition (*n* = 4 for fed and fasted group, respectively). Note the dramatic population changes and neuronal activation of AgRP/POMC neurons in the fasted ARC region, rapidly and easily identified by only simple immersion in AICI. * *p* < 0.05, ** *p* < 0.1.

AICI is useful not only for the high-resolution identification of neuronal structures, but also for the examination of neuronal distribution and projection in deep tissue. To exemplify this, we imaged the distribution of AgRP/POMC neurons in the hypothalamic region of mouse brains (rostral to caudal) expressing agouti-related peptide (AgRP)-tdTomato and pro-opiomelanocortin (POMC)-EGFP. The samples were imaged after 1 h of AICI incubation. Previous reports have generally examined neuronal distribution for a large field of the brain through many numbers of sections using a long imaging time and a complex process. However, we were able to obtain rapidly and easily for the 3D distribution of AgRP/POMC neurites in the hypothalamic region using AICI within 1 h (Figure 6A). Most AgRP neurons are located in the ventromedial region (VMH) of the arcuate hypothalamic nucleus (ARC) adjacent to the third ventricle, whereas POMC neurons are predominantly located dorsal and lateral to the AgRP neurons of the ARC (Figure 6B). The cell bodies of the AgRP neurons in the supraoptic nucleus (SO) were densely located in the periventricular hypothalamic nucleus (Pe) adjacent to the third ventricle in the dorsal area. We were able to follow the neurite projections to the ventral area of the hypothalamic ARC. The neuronal cell bodies of POMC are mostly located in the retrochiasmatic area (RCh), and the ARC and their neurites project to the preotic area of the hypothalamus and lateral third ventricle area (Figure 6C and Supplemental Movie S1).

We further examined whether AICI can be used for functional analysis with a fasting-induced neuronal activation animal model. AgRP and POMC neurons in the hypothalamic ARC are the key regulators of food intake and energy homeostasis [37,38,39]. Each AgRP neuron is independently activated, and projects to various nuclei of the hypothalamus and other brain regions [37]. AgRP neurons are activated in fasting mice, and c-Fos expression is increased in activated AgRP neurons [40,41]. Thus, we followed the c-Fos expression patterns in AgRP/POMC neurons to identify the multifunctional changes in 18 h fasting-induced energy-deficit states. The c-Fos expression in the ARC was increased in the AgRP neurons and predominantly colocalized with the AgRP neurons, whereas these changes were not observed in the POMC neurons as previously reported [42] (Figure 6D). c-Fos expression did not increase in the VMH, and the population of AgRP neurons was increased in the hypothalamic ARC in the fasting animals, whereas the population and intensity of POMC neurons decreased upon fasting (Figure 6E–G). The 3D images obviously show c-Fos expression, colocalization, population, and different neurite densities of AgRP/POMC neurons in the ARC (Figure 6H,I). AICI allowed rapid and easy multi-channel, three-dimensional imaging of deep brain tissues that permitted an analysis of the functional roles of AgRP/POMC neurons in the ARC during fasting at time points within 10 h.

## 3. Discussion

Tissue clearing methods have recently been developed that prevent protein denaturation, fluorescence quenching, size changes, and morphological distortion in samples. Nevertheless, there are still many limiting factors for imaging, including light scattering by cellular components, tissue density, and tissue hardness. Various factors, including light scattering, light absorption, and autofluorescence in biological tissues, interfere with obtaining high-resolution 3D images from thick tissue. Thus, all tissue clearing methods use the RI matching solution as the last procedure to reduce anomalies in imaging. However, most RI matching solutions for clearing samples have high viscosity, which consequentially causes issues including difficulty in handling samples and the formation of air bubbles during sample preparation [1].

Here, we developed AICI as a new water-based rapid optical clearing and RI matching method that is compatible with the 3D imaging of deep tissue and various fluorescent proteins, including staining dyes and fluorescence-conjugated antibodies. AICI has several advantages, such as rapid tissue clearing with simultaneous RI matching, application for use in imaging thick brain tissue, a low viscosity that is similar to water, little sample distortion, non-toxicity, and a simple protocol compared with previously described tissue clearing methods. AICI can be directly applied to post-fixed tissue with 4% PFA, and can easily clear tissue even at room temperature without any equipment. It also leads to only relatively small morphological expansion and distortion (Figure 2 and Supplemental Figure S1). The main advantage of AICI is that it is a low-viscosity miscible solution, close to the viscosity of water. Hence, it is possible to take 3D images of deep tissue with LSFM and confocal microscopy without image distortion caused by air bubbles and for long periods of imaging. AICI also clears the tissue of other organs, such as intestine, lung, heart, liver, kidney, and spleen. The spleen has particularly high levels of hemoglobin from red blood cells, which absorb light making imaging difficult. AICI and MS were able to clear spleen tissue samples upon incubation at 35 °C for 36 h, whereas EasyIndex and XclarityMS did not clear this tissue, even after a 48 h incubation (Supplemental Figure S4). Figure 5 and Figure 6 show that the AICI clearing method can take high-resolution images of fine neuronal structures such as dendritic spines and axonal boutons. Moreover, it allowed imaging of brain tissue that was 3 mm thick and incubated for 3 days. Thus, AICI can be applied to research in a wide range of fields, including neuroscience, developmental biology, and even cancer biology, and is particularly advantageous for microcircuit studies using deep brain tissue that is less than 3 mm thick from fluorescence protein-expressing transgenic mice or other model systems.

Indeed, we attempted to simultaneously determine the hypothalamic microcircuit and functional activity of POMC neurons and AgRP neurons under fasting-induced energy-deficit conditions using the AICI method (Figure 6). Previous reports indicate that hypothalamic neuronal circuits are dramatically altered in morphology during the control of body homeostasis; for example, under dehydration and during lactation [43,44]. Both the POMC and AgRP neuronal populations of the hypothalamic ARC are key regulators of food intake, and play opposing roles in food starvation [45]. AgRP gene expression and the dendritic spine and synapse density in the ARC were increased by fasting [42,46], and POMC neurons in the ARC are inhibited under fasting conditions [40,47,48]. ARC neurons innervate the paraventricular nucleus (PVN), and AgRP neurons in the ARC also predominantly project to the PVN [49].

We introduce here for the first time a 3D multimodal imaging approach for hypothalamic POMC and AgRP neurons. The AICI immersion method allowed us to show the diverse effects of the hypothalamic POMC and AgRP neurons by fasting using fluorescence-expressed transgenic mice and rapid immunostaining. First, we identified the hypothalamic POMC and AgRP neuronal distribution in the rostral to caudal hypothalamus. Consequently, the population and neurite density of AgRP neurons in the hypothalamic ARC were greatly increased by fasting compared with the fed animal group, as previously reported. Second, the neuronal intensity of POMC neurons in the ARC was decreased by fasting, whereas the population of POMC neurons did not significantly change. Third, we attempted to identify the functional effects of hypothalamic AgRP and POMC neurons on c-Fos expression in each neuron. The expression of c-Fos was significantly increased in the ARC by fasting and was highly colocalized with AgRP neurons, whereas it rarely colocalized with POMC neurons, even in the fasting animal. These results suggest that, of all AgRP neurons in the ARC, c-Fos-expressing AgRP neurons may function during long-time starvation. It is also possible that these neurons project to specific brain regions, although further studies are needed to clarify this. AgRP neurons project to the various brain regions associated with gamma-aminobutyric acid (GABA) release [50], and each of the target regions receives inputs from a separate subset of AgRP neurons [37,51]. Recent papers indicate that the direct projection of AgRP neurons to the medial preoptic area (mPOA) can modulate maternal nest-building [52]. These data suggest that POMC and AgRP neurons in the ARC are sensing and responding differently to energy deficits during fasting, and that the alteration of neuronal population and neurite density may be linked to specific behavior.

Taken together, AICI can be applied to the analysis of neuronal microcircuits linked to various animal behaviors using tissues expressing multiple factors that are fluorescently marked. This study has limitations, as we only compared AICI to three commercial RI matching solutions rather than other simple immersion methods, such as high-concentration solutions of sucrose and fructose, glycerol, TDE, or an ultrafast optical clearing method (FOCM). However, these simple immersion methods using sucrose, fructose, and glycerol require other clearing steps, including using different concentrations of replacement solution, and have issues such as sample shrinkage and high viscosity [3,23,30]. The FOCM has few limitations, recommending 20–300 µm thickness of tissue for slice clearing, and three days for incubation in FOCM solution at 25 °C for hemibrain clearing, although it can be used for the ultrafast clearing of fixed brain samples [53]. OCAs, including sugar, fructose, glycerol, TDE and contrast agents, have been used as RI matching solutions for various clearing methods, despite several limitations. Thus, we compared several factors, such as optical clearing, imaging depth, sample distortion, viscosities for 3D imaging, and fluorescence preservation, using a commercially available immersion medium that is based on these OCAs.

In summary, our results indicate that AICI can rapidly penetrate and easily clear brain tissues up to 3 mm-thick using aqueous OCAs, and can allow microcircuits in small brain regions to be imaged through improved optical clearing and 3D imaging. Moreover, AICI can be applied to antibody and dye-stained brain tissues, and its low viscosity (close to that of water) allows confocal microscopy and LSFM imaging while minimizing the effect of air bubbles. Therefore, AICI can be widely applied to the imaging of biological tissue with simple immersion. This method will help researchers to study the complex structures of large and thick organ tissue samples via an easily made, low-cost clearing and RI matching solution.

## 4. Materials and Methods

### 4.1. Mice

Genetically modified Tg(Thy1-EGFP)MJrs/J mouse line were kindly provided by Dr. Rah’s lab in KBRI and were originally purchased from Jackson Laboratories (JAX stock #007788, Bar Harbor, ME, USA ) and bred in-house. Tg(Thy1-EGFP)MJrs/J mice were genotyped with a primer set from JAX protocol [36]. Genotyping of Thy1-EGFP mice was confirmed using primers 5′-CGGTGGTGCAGATGAACTT-3′ and 5′-ACAGACACACACCCAGGACA-3′ for 415 bp, 5′-GTAGGTGGAAATTCTAGCATCA-TCC-3′ and 5′-CTAGGCCACAGAATTGAAAGATCT-3′ for a 324 bp internal positive control band. POMC-EGFP/AgRP Cre-tdTomato mice were kindly provided by Dr. Kim’s lab from Incheon National University. Transgenic mice (POMC-EGFP mice; stock #009593, AgRP-Cre mice; stock #012899 and Tdtomato mice; stock #007914) were obtained from the Jackson Laboratory. The tdTomato reporter mice were crossbred with AgRP-cre mice to label AgRP-positive neurons with tdTomato signals. To further generate transgenic lines showing double fluorescence signals in AgRP and POMC neurons, POMC-EGFP mice were crossbred with AgRP Cre-tdTomato mice. Genotyping for transgenic mice was confirmed using primers from Jackson Laboratory: POMC-EGFP, 5′-AAGTTCATCTGCACCACCG-3′ and 5′-TCCTTGAAGAAGATGGTGCG-3′ for 173 bp; AgRP-Cre, 5′-GCTTCTTCAATGCCTTTTGC-3′ and 5′-AGGAACTGCTTCCTTCACGA-3′ for 280 bp; tdTomato, 5′-GGCATTAAAGCAGCGTATCC-3′ and 5′-CTGTTCCTGTACGGCATGG-3′ for 196 bp. For sampling, male and female mice between 6–8 weeks of age were deeply anesthetized with Avertin (2.5%, Tribromoethanol, intraperitoneal(i.p.)) in 1x PBS and transcardially perfused with ice cold PBS to flush the blood vessels, and then 4% paraformaldehyde (PFA) in PBS. The fixed brain was postfixed with 4% PFA in PBS at 4 °C overnight. Each fixed brain was washed with PBS, and brain slices were prepared using the coronal brain matrix. Mice were housed in groups of 2–5 animals per cage with ad libitum access to standard chow and water in 12/12 light/dark cycle with “lights-on” at 07:00, at an ambient temperature of 20–22 °C and humidity (about 55%) through constant air flow. The well-being of animals was monitored on a regular basis. The experimental design was reviewed and approved by the Institutional Animal Care Use Committee (IACUC) of the KBRI (IACUC-18-00018, IACUC-20-00051).

### 4.2. AICI Reagent Preparation

For simple immersion clearing, AICI (Aqueous Immersion Solution for tissue Clearing and Imaging) reagent was prepared as 20% (wt/vol) *N*-methyl-D-glucamine (#M2004, Sigma-Aldrich, St. Louis, MO, USA), 30% (wt/vol) TDE (#166782, Sigma-Aldrich, St. Louis, MO, USA), 45% (wt/vol) Iodixanol (#D1556, Sigma-Aldrich, St. Louis, MO, USA), 0.2% (wt/vol) Triton X-100 (20%) (#T8787, Sigma-Aldrich, St. Louis, MO, USA), 0.5% (wt/vol) α-thioglycerol (#88640, Sigma-Aldrich, St. Louis, MO, USA), 1% (wt/vol) Triethanolamine (#90278, Sigma-Aldrich, St. Louis, MO, USA) dissolved in pure DW (#10977-015, Invitrogen, Carlsbad, CA, USA). After mixing with a magnetic stirrer for 2 h at 25 °C, the solution was filtered using a bottle-top vacuum system into an amber bottle. The reagent was stored for several months at 25 °C.

### 4.3. Transparency and Linear Expansion of Brain Slice

A fixed Thy-1-GFP mouse brain was cut into 1 mm slices on mouse brain coronal matrix. Commercialized RI matching mediums such as Easyindex (LifeCanvas Technologies, Cambridge, MA, USA), XclarityMS (Logos Biosystems, Anyang-si, Korea), MS (Binaree Inc., Daegu, Korea) and AICI were prepared in a separate well on the well-plate, and a 1 mm slice of brain was placed into each well. Subsequently, the well-plate was rotated by two pathways at 25 °C and 35 °C in incubator. Each of the RI medium treated brain slices was captured at specific intervals to compare its transparency and expansion during clearing. Relative areas were measured by ImageJ in the resulting images. Normalized expansion ratio of the initial state to the final distortion (at 36 h) was calculated for quantification.

### 4.4. Transmission Measurements

The light transmittance of different RI matching solutions and cleared 1 mm brain slice tissues in a 24-well plate was calculated using a multimode microplate reader (FlexStation 3, Molecular Devices, San Jose, CA, USA). Light was illuminated on the gray matter of the cortex to measure the absorbance of the brain slices, as light scattering is different for the brain slice region. The absorbance (A) was measured in each RI matching solution from 400 to 800 nm with a 25 nm bandwidth. The absorbance was converted to transmittance (%) using the Lambert–Beer law equation. Sample expansion can reduce light scattering; thus, we used samples of each RI matching solution after 24 h of incubation, the period of time in which expansion or shrinkage almost stops in the different OCA-based RI matching solutions.

### 4.5. Hardening and Viscosity Measurement

Reagents for clearing were exposed at room temperature 25 °C for 36 h, and capillary phenomena for each reagent was compared. The well-plate was tilted to observe the hardening process of the reagents over 30 days. Viscosities of all reagents were measured by Haake Mars rheometer (ThermoFisher Scientific, Waltham, MA, USA) with a 35 mm rotor set on the 25 °C bottom plate. The viscosity was measured in the shear rate in the scope of 0.01 to 10 $s^{-1}$. The experiment was controlled by Haake RheoWin Job manager (ver 4.70, ThermoFisher Scientific, Waltham, MA, USA).

### 4.6. Durability of Reagents for Imaging

To identify the durability of viscosity for each reagent, cultured HEK293T cells in a 24 well-plate format were fixed with 4% PFA. A non-reagent treated image of the cells was acquired. Each well was then treated with RI matching reagents and the same region was captured every 5 min overnight at 25 °C.

### 4.7. 3D-Imaging of Brain Slices Treated with AICI

The periphery of hippocampus was coronally sectioned into slices of 1–3 mm-thickness for 3D imaging. After treating the slices for different lengths of times according to their thickness, they were embedded in 2% agarose, trimmed, put into AICI reagent again, and placed on the shaker for 1–2 h at 25 °C. The excitation source was a λ = 488 nm laser for GFP detection. 3D-images of brain slices were taken by light sheet fluorescence microscopy (LSFM, Lightsheet Z.1, Carl Zeiss, Germany). The illumination lens was a 5x, 0.1 NA at air and the objective of emission part was a 5x, 0.16 NA, EC Plan-Neofluar by Zeiss. The stitched 3D-images were 3 × 3 tiles with field of view of 2.47 × 2.47 mm (1920 × 1920 pixel), z-step size was a 5 μm. All acquisitions were controlled by ZEN (Carl Zeiss) software. The experiment for fine neuronal structure preserving was implemented by confocal microscopy (A1Rsi, Nikon, Tokyo, Japan) under the control of NIS (Nikon) software ver 5.0. The objective was a 60x, Plan Apochromat from Nikon. A field of view of images was a 212 × 212 μm (1024 × 1024 pixel) with 0.2 μm z-step varying focus depth.

### 4.8. Immunostaining for Multifunctional Imaging by AICI

POMC-EGFP/AgRP Cre-tdTomato double fluorescence expressing mice were cardiac perfused with 4% PFA and the hypothalamus transversally cut to a thickness of 700 μm using the Leica VT100 S vibratome. For immunostaining, the sliced brain was incubated for 2 h with blocking solution (10% Normal horse serum and 2% TritonX-100 in PBS) and washed on a gentle shaker twice for 30 min. Subsequently, mouse brain slices were stained using the electrophoretic immunostaining system (C-stain, Crayon technologies, Gyeonggi-do, Korea) with the following antibodies: rabbit anti-c-Fos (1/200, Cat# 2250S, Cell Signaling, Danvers, MA, USA) for 2 h and washed for 30 min three times with a large volume of washing buffer. Washed brain slices were then processed in the C-stain system again with a Goat anti-Rabbit Alexa fluor 647 (1/500, Cat# A-21245, Invitrogen, Carlsbad, CA, USA) for 2 h and washed for 30 min three times as with the primary antibody washing.

### 4.9. Stitching and 3D-Rendering of the Stack Images

Images of multiple regions were stitched manually with Imaris Stitcher software (Ver 9.2.1, Bitplane), due to the relatively large size of the sample, using LSFM. Both LSFM images and fine structure stack images of confocal microscopy were rendered using Imaris software (ver 9.2.1, Bitplane).

### 4.10. Statistical Analysis

Analysis and quantification of data were performed with SigmaPlot 14.0 (Systat Software, Point Richmond, CA, USA), and data are presented as means ± SEM. Results were analyzed to group and times using one-way ANOVA, and differences were considered significant when *p* < 0.05. Significance is indicated as follows: * *p* < 0.05; ** *p* < 0.01; *** *p* < 0.001; or N.S., not significant.

## Data Availability

Not applicable.

## Supplementary Materials

The following supporting information can be downloaded at: https://www.mdpi.com/article/10.3390/ijms23126826/s1.

## Abbreviations

RI	Refractive index
WD	Working distance
PFS	Perfect focus system
ARC	Hypothalamic arcuate nucleus
AgRP	Agouti-related peptide
POMC	Pro-opiomelanocortin
ARH	Arcuate hypothalamic nucleus
VMH	Ventromedial hypothalamic nucleus
MPO	Medial preoptic nucleus
LHA	Lateral hypothalamic area
ME	Median eminence
TU	Tuberal nucleus
OCA	Optical clear agent
Pe	Periventricular hypothalamic nucleus
RCh	Retrochiasmatic area
SO	Supraoptic nucleus
PVN	Paraventricular nucleus
GABA	Gamma-Aminobutyric acid
FOCM	Ultrafast optical clearing method
