# Supplementary material for: Single-Step Fast Tissue Clearing of Thick Mouse Brain Tissue for Multi-Dimensional High-Resolution Imaging
Source: Int J Mol Sci. 2022 Jun 19;23(12):6826. doi: 10.3390/ijms23126826 (PMC9224586; doi:10.3390/ijms23126826)
Supplement: Supplementary file 1 [file ijms-23-06826-s001.zip › ijms-1755039-supplementary.pdf]

Supplemental Figure Legends

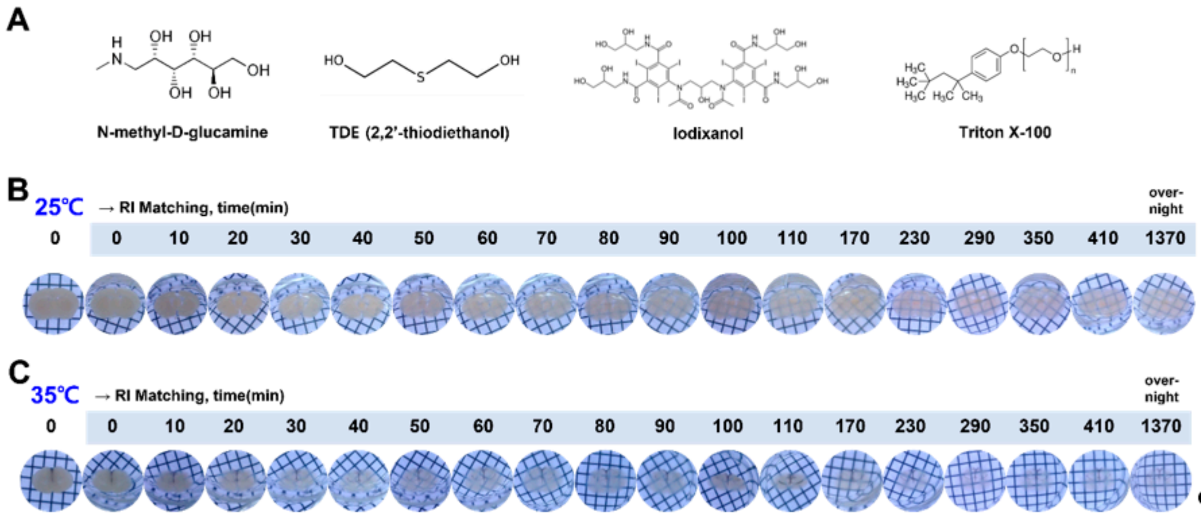

Supplemental Figure S1. AICI rapidly diffused and clarified in 1 mm thick brain slices. (A) Major chemical components of AICI. (B and C) Changes in transparency of brain slices by AICI over time at indicated temperatures (25 °C, 35 °C). The degree of clearing at 35 °C was faster than that of clearing at 25 °C in 1 mm thick brain slices.

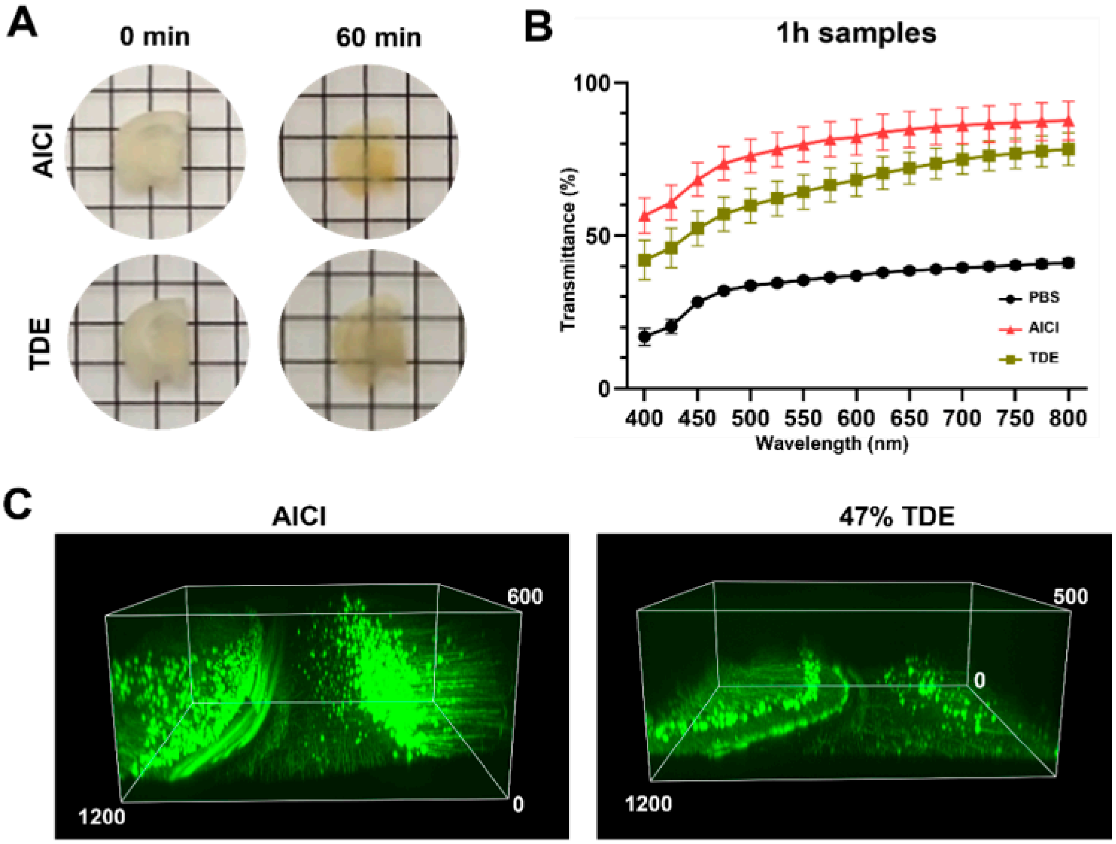

Supplemental Figure S2. Comparison of tissue transparency in AICI with TDE-based RI matching solution. (A) Representative images of tissue transparency at 35 ℃ in AICI and 47% TDE-based RI matching solution, respectively. The 1mm thick coronal brain slices from Thy1-EGFP mice were treated with both RI matching solutions for 1 h. (B) Transmission curves of each RI matching solution and 1X PBS (Mean ± SEM, n = 3). (C) Comparison of 3D volume images of Thy1-EGFP mouse brain slices (cerebral cortex) by high-resolution confocal imaging, brain slices were treated with AICI (left panel) and 47% TDE (right).

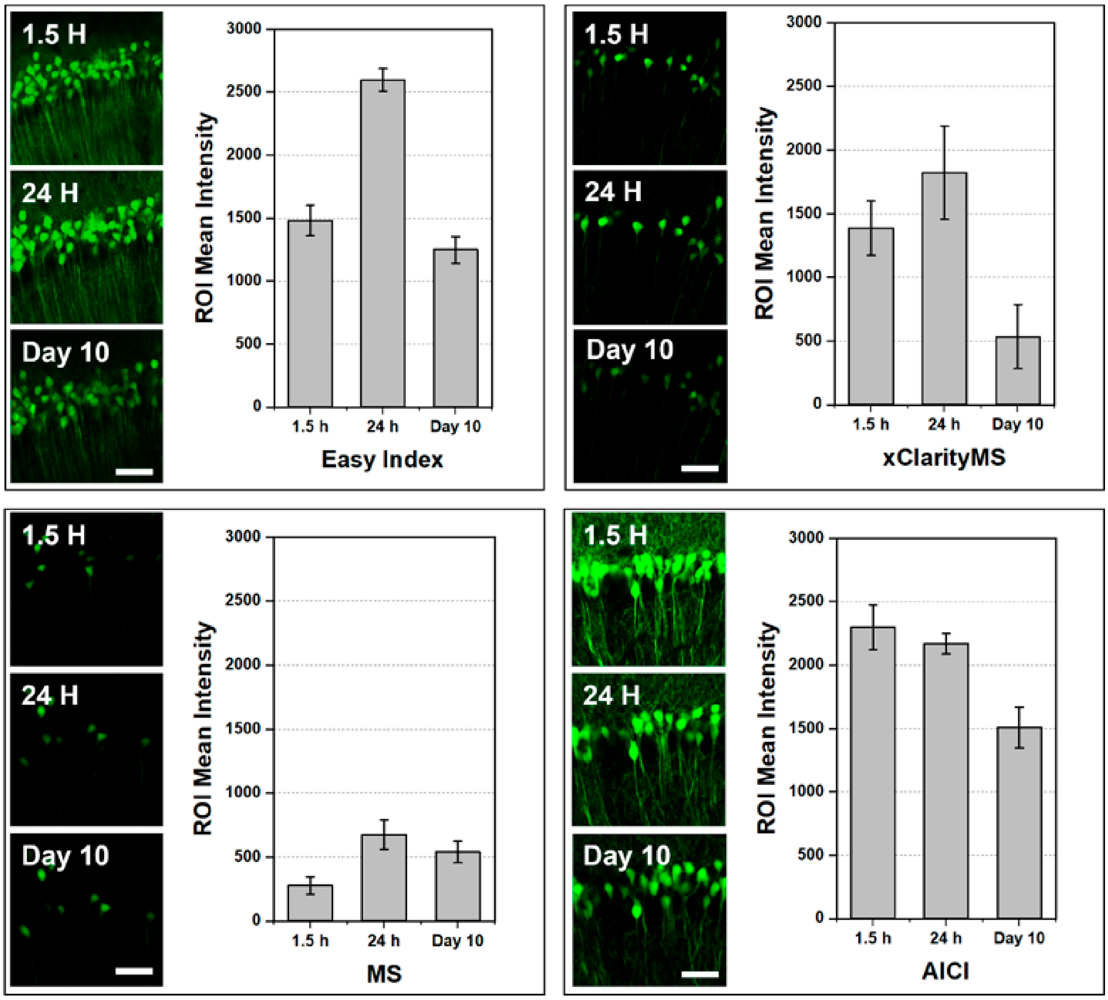

Supplemental Figure S3. Fluorescent intensity preservation effect was observed after different reagent-based RI matching solution incubation for 10 days. The 1 mm brain slices from Thy1-GFP-M mice were divided into quarter regions and fluorescence intensity was measured for each neuronal cell body of a cortex single image at a 50 μm depth using confocal microscopy. The left image of each panel represents fluorescence intensity after each RI matching solution incubation under the same conditions. Fluorescence intensity was calculated for each cell body (n = 30) and the experiment was repeated three times. Scale bar, 50 μm.

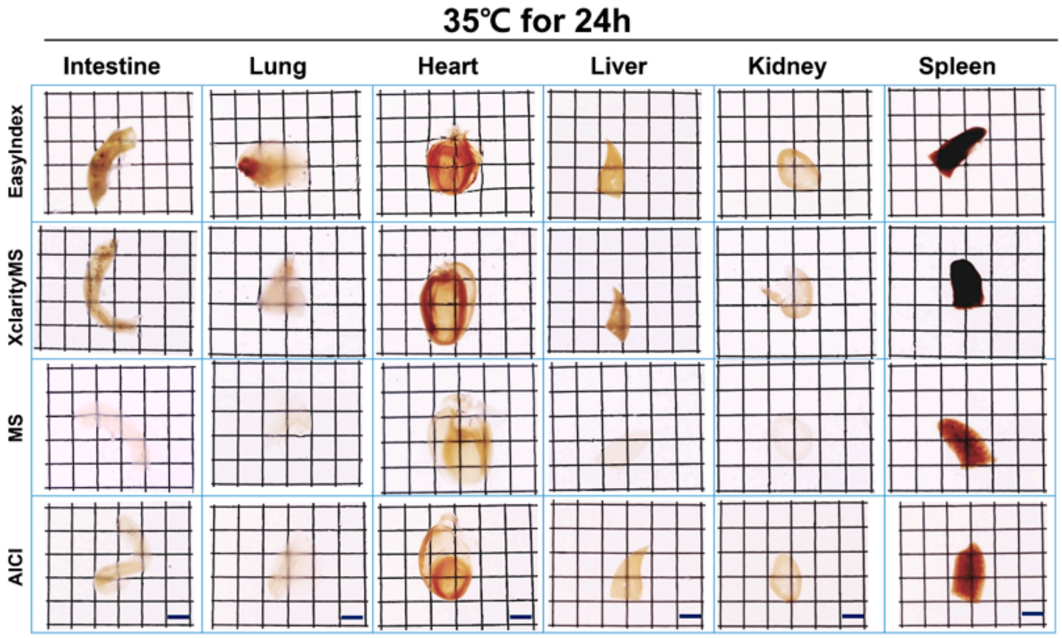

Supplemental Figure S4. Optical clearing effects in various organs using commercially available refractive index matching medium and AICI. Organs were perfused with 4% PFA and part of the organ tissues were incubated in each indicated RI medium for 24 h at 35 ℃. Liver and kidney were sectioned to 1 mm tissue slices and the heart was transversely sectioned covering half the tissue. Scale bar, 3 mm.
