# Supplementary figures and images for: Single-Step Fast Tissue Clearing of Thick Mouse Brain Tissue for Multi-Dimensional High-Resolution Imaging
Source: Int J Mol Sci. 2022 Jun 19;23(12):6826. doi: 10.3390/ijms23126826 (PMC9224586; doi:10.3390/ijms23126826)

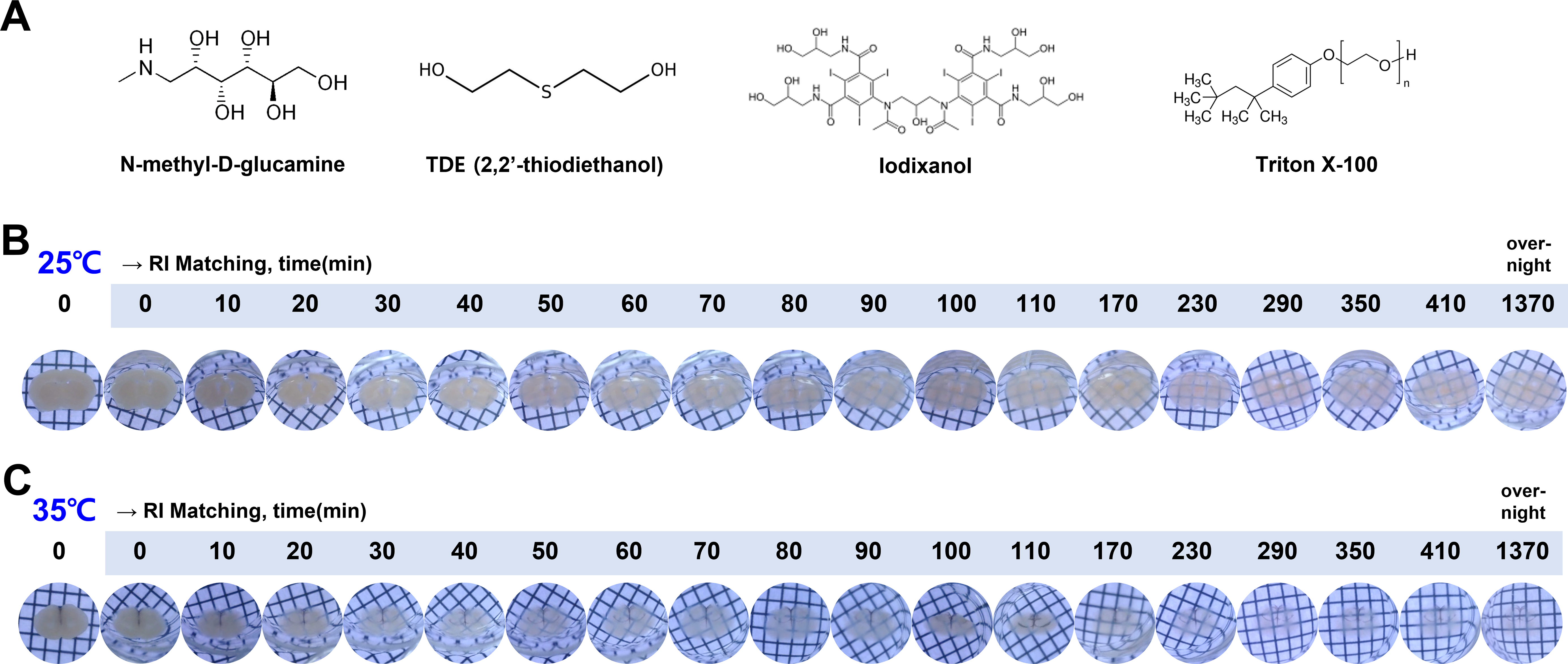

Supplement: Supplementary file 1 [file ijms-23-06826-s001.zip › Supplemental Figure S1.tif]

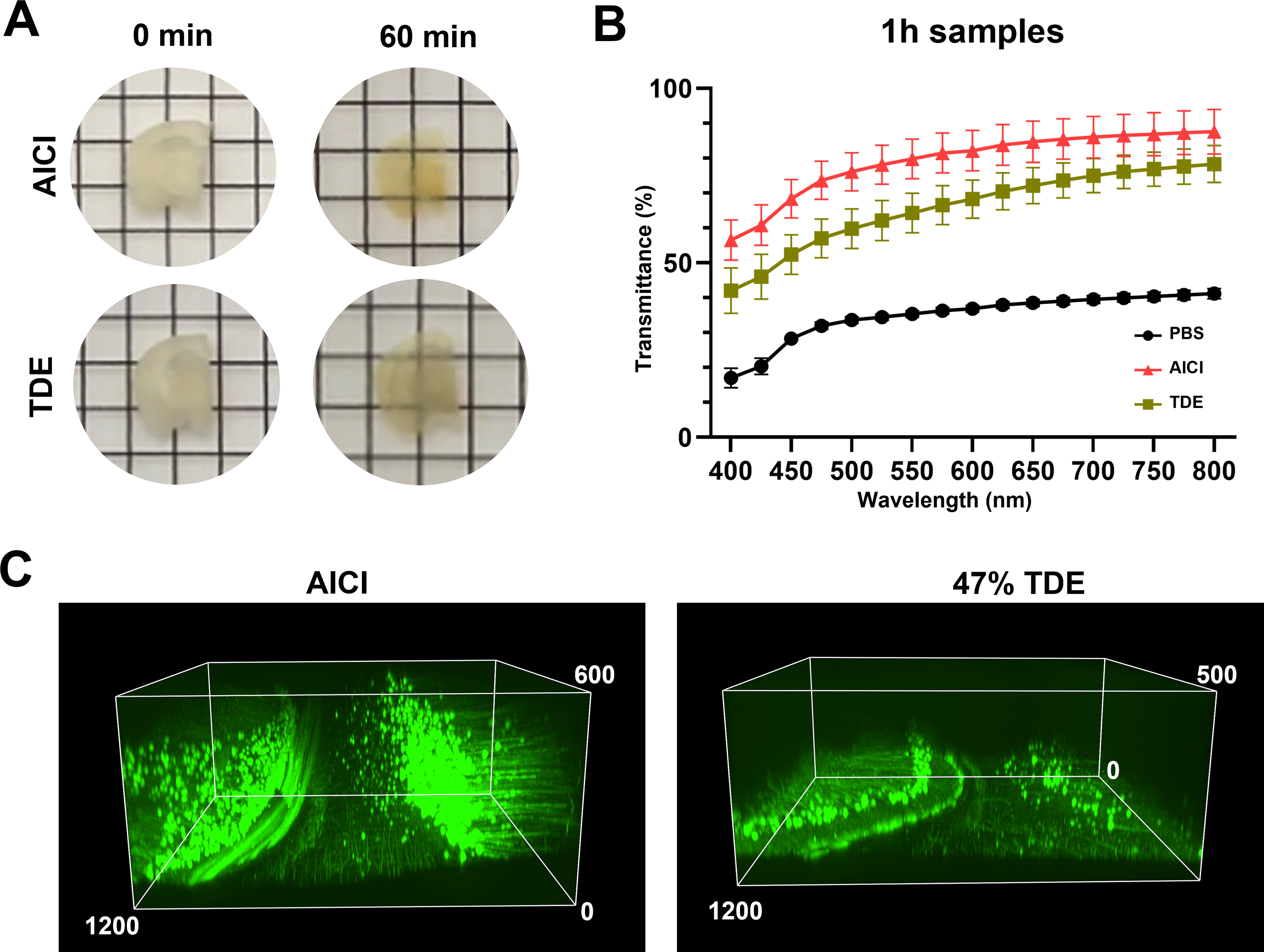

Supplement: Supplementary file 1 [file ijms-23-06826-s001.zip › Supplemental Figure S2.tif]

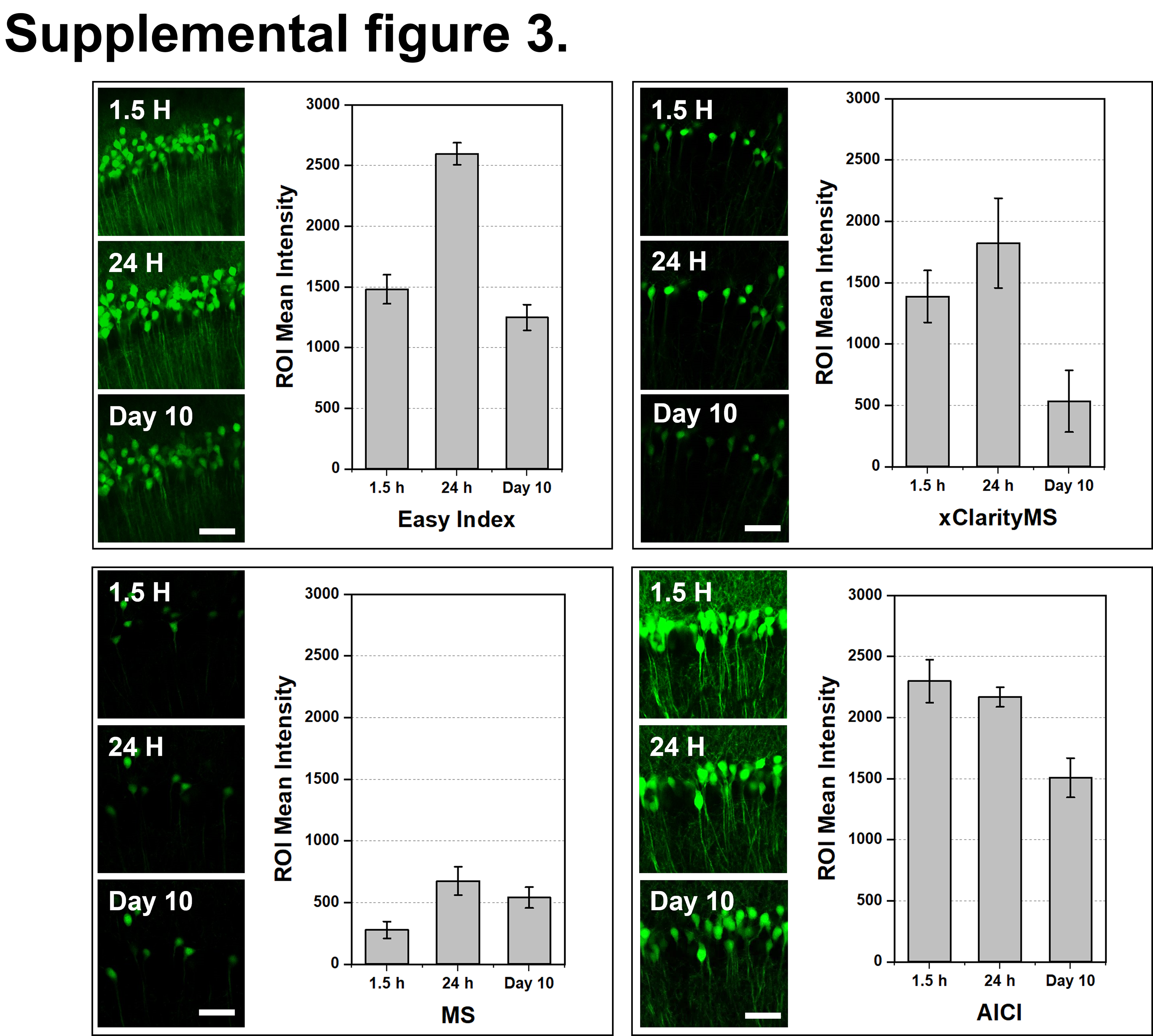

Supplement: Supplementary file 1 [file ijms-23-06826-s001.zip › Supplemental Figure S3.tif]

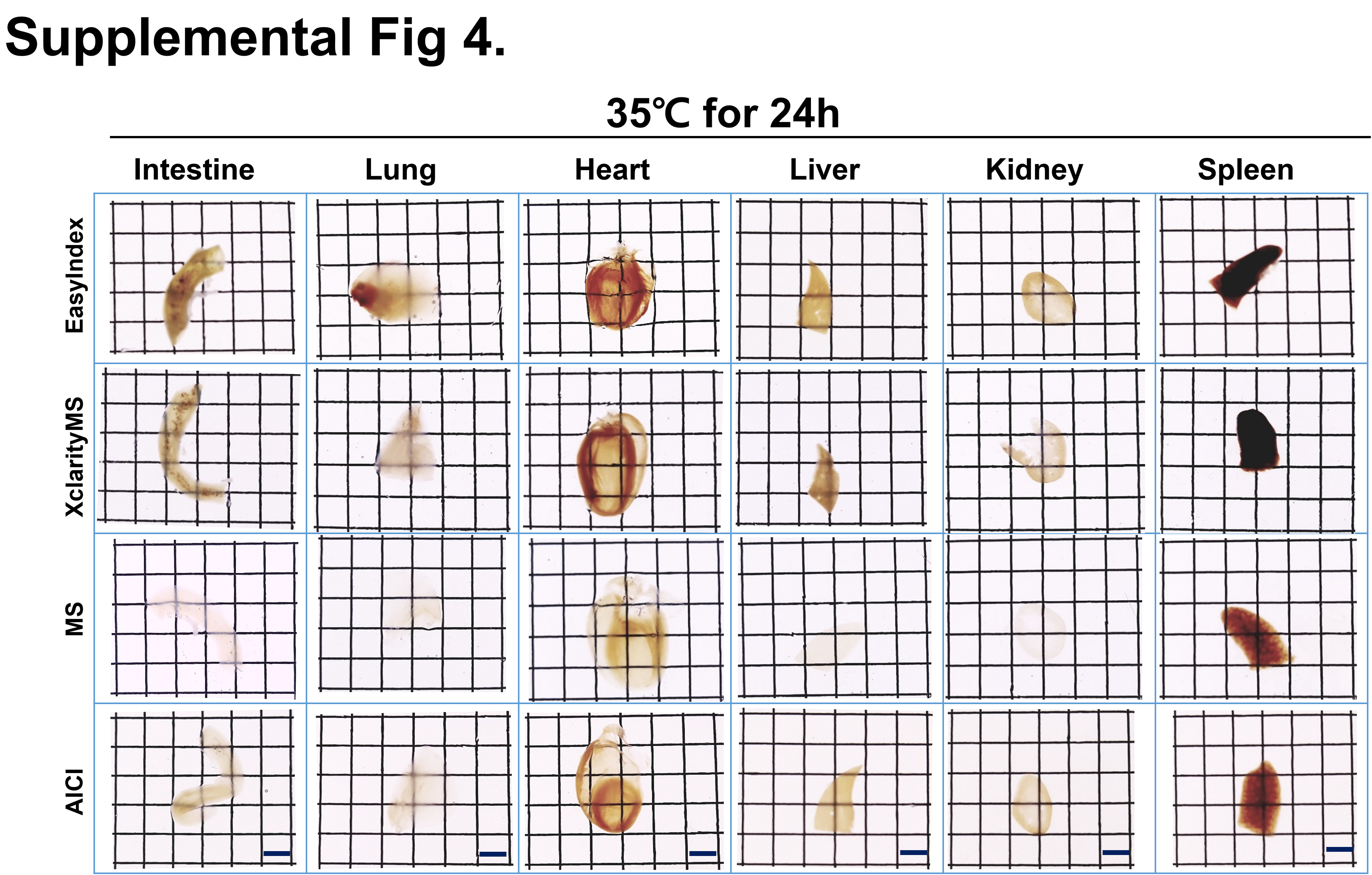

Supplement: Supplementary file 1 [file ijms-23-06826-s001.zip › Supplemental Figure S4.tif]
